# Gender differences in pain levels before and after treatment: a prospective outcomes study on 3,900 Swiss patients with musculoskeletal complaints

**DOI:** 10.1186/1471-2474-13-241

**Published:** 2012-12-05

**Authors:** Cynthia K Peterson, B Kim Humphreys, Jürg Hodler, Christian WA Pfirrmann

**Affiliations:** 1 Department of Radiology and Department of Chiropractic, Orthopaedic University Hospital of Balgrist, Forchstrasse 340, 8008, Zürich, Switzerland; 2 Department of Chiropractic, Orthopaedic University Hospital of Balgrist, Forchstrasse 340, 8008, Zürich, Switzerland; 3 Department of Radiology, University Hospital, Rämistrasse 100, 8091, Zürich, Switzerland; 4 Department of Radiology, Orthopaedic University Hospital of Balgrist, Forchstrasse 340, 8008, Zürich, Switzerland

**Keywords:** Gender differences, Pain intensity, Sex differences, Outcomes, Pain

## Abstract

**Background:**

Current studies comparing musculoskeletal pain levels between the genders focus on a single point in time rather than measuring change over time. The purpose of this study is to compare pain levels between males and females before and after treatment.

**Methods:**

Eleven different patient cohorts (3,900 patients) included in two prospective outcome databases collected pain data at baseline and 1 month after treatment. Treatments were either imaging-guided therapeutic injections or chiropractic therapy. The Mann–Whitney *U* test was used to calculate differences in numerical rating scale (NRS) median scores between the genders for both time points in all 11 cohorts.

**Results:**

Females reported significantly higher baseline pain scores at 4 of the 11 sites evaluated (glenohumeral (p = 0.015), subacromial (p = 0.002), knee (p = 0.023) injections sites and chiropractic low back pain (LBP) patients (p = 0.041)). However, at 1 month after treatment there were no significant gender differences in pain scores at any of the extremity sites. Only the chiropractic LBP patients continued to show higher pain levels in females at 1 month.

**Conclusions:**

In these 11 musculoskeletal sites evaluated before and after treatment, only 3 extremity sites and the chiropractic LBP patients showed significantly higher baseline pain levels in females. At 1 month after treatment only the LBP patients had significant gender differences in pain levels. Gender evaluation of change in pain over time is likely to be more clinically important than an isolated pain measurement for certain anatomical sites.

## Background

While everyone with an intact nervous system experiences physical pain, research has shown that the reported intensity and prevalence of pain arising from the musculoskeletal system appears to differ between males and females [1,2]. Specifically, there is a higher prevalence of pain in females suffering from headaches, neck and back pain, as well as knee pain, and this increased prevalence starts during adolescence [1]. A recent report from the United States also suggests that pain is under diagnosed and under treated in females [3]. Several studies focusing on musculoskeletal (MSK) pain and gender report that females are more likely to experience chronic pain, more likely to receive treatment for chronic pain compared to males, and report significantly higher pain intensity scores [4-6]. What remains unknown is the reason for these higher prevalence rates of pain in females. Do they reflect differences between males and females in willingness to seek medical care as has been suggested in previous research [1]?

Two recent systematic reviews of the literature looking at these gender differences in an experimental environment concluded that males have significantly more efficient diffuse noxious inhibitory controls (DNIC) compared to females and females are less tolerant to thermal stimuli and pressure pain, but not to ischemic pain. But the weight of the evidence does not show a clear and consistent pattern of gender differences in pain sensitivity [7,8]. However, all studies in these systematic reviews were conducted in the laboratory setting and it is unknown how closely these results reflect what is seen in daily clinical practice. Although it has been suggested that pain management should take these baseline gender pain differences into account [1], it has yet to be shown that higher baseline pain levels indicate a worse response to treatment. Current studies compare reported pain levels between males and females at a single point in time rather than assessing changes over time [1,2,4-8].

This specialized orthopaedic/rheumatology university hospital began collecting prospective outcomes data during 2009–2010 and this is ongoing in both the radiology and chiropractic departments in order to create large databases for research and quality assurance purposes. Patients undergoing imaging-guided therapeutic musculoskeletal injections and patients being treated by chiropractors for neck pain, low back pain, and imaging confirmed symptomatic disc herniations are included in these databases. Because the literature consistently reports gender differences in pain levels and prevalence based on anatomical region [1-6], assumptions have been made about how this should influence treatment [1]. Therefore the purposes of this study were to first assess gender differences in baseline pain levels based on anatomical site in patients of this specialized orthopaedic/rheumatology hospital and second whether any baseline gender differences in pain levels continued 1 month after the start of treatment.

## Methods

This is a prospective outcomes study with 1 month follow-up on 11 different cohorts of patients using two large databases, one from the radiology department and the other from the chiropractic department. The study was conducted in a specialized orthopaedic/rheumatology university hospital. Ethics approval was obtained from the Canton of Zürich ethics commission prior to the start of the databases (EK-12/2009, EK-16/2009, EK-19/2009, EK-22/2009) and all patients signed informed consent immediately prior to their therapeutic intervention. No patient was included in any of the databases more than once.

### Imaging-guided musculoskeletal injections patient Selection

Consecutive patients over the age of 18 with pain strongly suspected to arise from the particular joint, nerve root or spinal canal targeted for injection and who had not responded to conservative treatments were included. Exclusion criteria included joint injections not intended for pain therapy (i.e. arthrography, joint aspiration for infection or biopsy). Patients with overlying skin infections or who were pregnant were also excluded. Patients on anticoagulants were excluded from having spine injections but not from the peripheral joint injections.

Only those imaging-guided injection sites with at least 100 patients in the current database were included for analysis in order to limit this study to the most common MSK injection sites. This included cervical indirect nerve root blocks, lumbar epidural injections, lumbar facet joint injections, and lumbar nerve root blocks for the spine. Extremity injection sites included the gleno-humeral, subacromial, knee and hip joints.

### Chiropractic treatment patient selection

New patients over the age of 18 with back pain, neck pain, or magnetic resonance imaging (MRI) confirmed symptomatic lumbar disc herniation of any duration who had not received chiropractic or manual therapy in the prior 3 months were recruited from multiple chiropractic practices in Switzerland. Patients with specific abnormalities of the lumbar spine that are contraindications to chiropractic manipulative treatment, including tumors, infections, inflammatory spondylo-arthropathies, acute fractures, Paget’s disease and severe osteoporosis were excluded.

Only the treatment sites with a minimum of 100 patients in the database were included in this study. Neck pain patients, low back pain patients, and patients with the specific MRI confirmed diagnosis of symptomatic lumbar disc herniation met this criterion. Patients in the cervical disc herniation database were not included due to the current small sample size.

### General imaging-guided injection protocol

**Figure 1:**
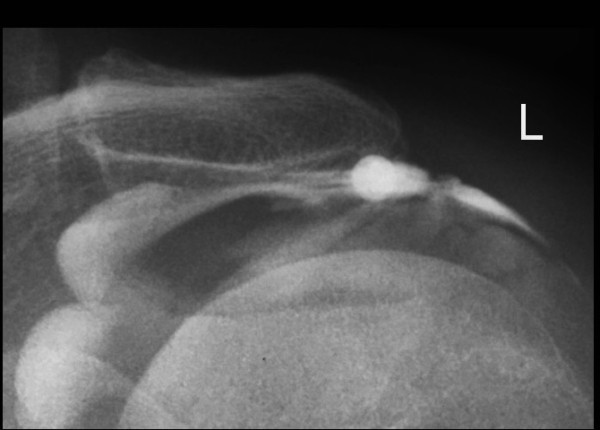
Imaging-guided subacromial injection showing contrast in the subacromial space.

**Figure 2:**
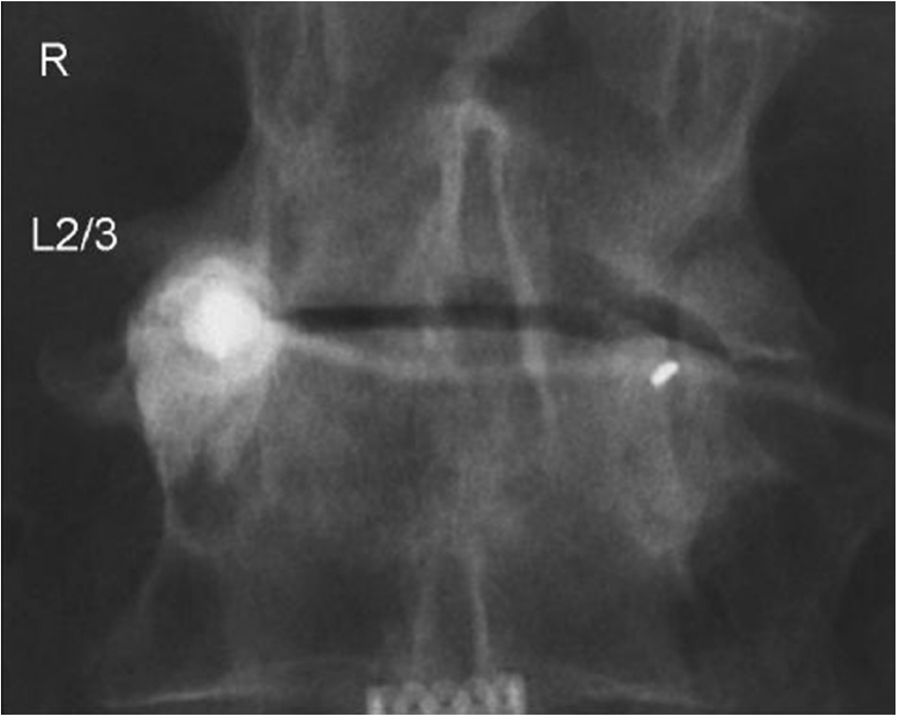
Imaging-guided right L2-3 facet injection showing the needle and contrast within the joint.

Under imaging guidance, a needle was placed into the relevant joint, adjacent nerve root or lumbar interlaminar space. A non-neurotoxic contrast agent was then injected to confirm correct needle placement. Once correct needle placement was confirmed, an injection of a local anesthetic (Naropin 0.2%) was delivered, followed by an injection of corticosteroid (Figures 1 and 2). The exact quantities of these injectates were determined by the specific joint or area being treated.

### Chiropractic treatments

Thirty two different Swiss chiropractors contributed patients to the chiropractic outcomes database and no individual data was collected concerning the specific treatments offered to each patient. However, data collected from the Swiss Job Analysis Study of chiropractors in 2009 [9] indicated that spinal manipulative therapy (SMT) using an approach known as the ‘Diversified’ technique was the most common treatment and applied to between 76 and 100% of patients. The other common additional treatments used included advice on the activities of daily living, trigger point therapy, therapeutic exercises and mobilization techniques.

### Data collection

The eleven point NRS (numerical rating scale) where 0 is no pain and 10 is the worst pain imaginable was used for all patients in the two databases. This value was recorded for the level of current pain in the radiology department immediately prior to the imaging-guided therapeutic injection or in the respective chiropractic clinics prior to the chiropractic treatment procedure. One month after the first treatment, patients who had received an imaging-guided therapeutic musculoskeletal injection returned a paper-based postal questionnaire which included information on their current pain level using the NRS scale. Chiropractic patients had this exact same data collected one month after the first treatment via telephone calls from trained independent research assistants. Data was manually entered into SPSS version 17.0 by one of the authors and one research assistant.

### Statistical analysis

Means and standard deviations (SD) were calculated for the two data collection time points by gender for each injection and chiropractfic treatment site. Because the NRS data was skewed toward the upper end of the pain scale, the non-parametric Mann–Whitney *U* test was used to assess for significant differences between the genders for baseline NRS, 1 month NRS, and the NRS change score median values at 1 month for each region. P < 0.05 was determined to be significant. However, the mean values rather than the median values are reported in the tables to make the data easier to relate to the NRS scale and for comparison with other prior studies. Spearman’s correlation coefficient was used to evaluate the relationship between age and baseline NRS scores for each of the 11 cohorts.

## Results

Data was available for 3,900 patients, 1,954 from the imaging-guided therapeutic injections database and 1,946 from the chiropractic treatment database. Of these patients 1,855 (48%) were males. Of the four spinal injection sites, there was a higher prevalence of females for both epidural (61%) and lumbar facet (55%) injections (Table 1). A slight predominance of male patients received cervical (55%) or lumbar (52%) nerve root injections. A higher prevalence of female patients was found in 3 of the 4 extremity injection sites. Women made up 52% of the gleno-humeral joint injections, 57% of the knee injection patients and 58% of the hip injection patients (Table 2). Fifty six percent of the subacromial injection patients were males.

**Table 1:** Gender comparisons for Pain with Spinal Injections

**PROCEDURE**	**DATA TIME POINT**	**MALES Mean (SD)**	**FEMALES Mean (SD)**	**P-VALUE**
**Cervical Nerve Root Injections (n = 132)**	*Baseline NRS*	6.27 (2.33) n = 73	6.94 (2.23) n = 59	0.998
	*1 Month NRS*	3.01 (2.96)	3.53 (2.86)	0.361
	*1 Month NRS Change score*	3.19 (2.93)	3.08 (2.74)	0.890
**Epidural injections (n = 165)**	*Baseline NRS*	6.12 (2.33) n = 65	6.55 (2.49) n = 101	0.492
	*1 Month NRS*	4.15 (3.01)	3.73 (2.60)	0.582
	*1 Month NRS Change score*	1.89 (2.67)	2.65 (3.20)	0.159
**Lumbar Facet injections (n = 424)**	*Baseline NRS*	6.53 (2.12) n = 191	6.79 (2.23) n = 233	0.148
	*1 Month NRS*	4.21 (2.55)	4.61 (2.65)	0.172
	*1 Month NRS Change score*	2.31 (2.58)	2.16 (2.72)	0.590
**Lumbar Nerve Root injections (n = 400)**	*Baseline NRS*	6.53 (2.27) n = 208	6.54 (2.23) n = 192	0.295
	*1 Month NRS*	3.75 (2.81)	3.79 (2.88)	0.611
	*1 Month NRS Change score*	2.74 (3.19)	2.70 (3.01)	0.994

n = number of patients. NRS = Numerical Rating Scale for pain. SD = standard deviation.

**Table 2:** Gender comparisons for Pain with Extremity Injections

**PROCEDURE**	**DATA TIME POINT**	**MALES Mean (SD)**	**FEMALES Mean (SD)**	**P-VALUE**
**Gleno-Humeral Injections (n = 224)**	*Baseline NRS*	5.79 (2.11) n = 107	6.51 (2.22) n = 117	0.015*
	*1 Month NRS*	2.72 (2.29)	3.00 (2.62)	0.791
	*1 Month NRS Change score*	3.03 (2.46)	3.51 (2.44)	0.112
**Subacromial Injections (n = 165)**	*Baseline NRS*	5.88 (2.08) n = 93	6.74 (1.75) n = 72	0.002*
	*1 Month NRS*	2.49 (2.19)	2.59 (2.32)	0.468
	*1 Month NRS Change score*	3.38 (2.88)	4.27 (3.01)	0.045*
**Knee Injections (n = 309)**	*Baseline NRS*	6.10 (2.58) n = 134	6.76 (2.08) n = 175	0.023*
	*1 Month NRS*	4.56 (2.88)	4.68 (2.95)	0.418
	*1 Month NRS Change score*	1.51 (2.80)	2.07 (2.96)	0.357
**Hip Injections (n = 135)**	*Baseline NRS*	5.78 (2.49) n = 57	6.47 (1.92) n = 78	0.387
	*1 Month NRS*	4.11 (2.75)	4.51 (2.79)	0.886
	*1 Month NRS Change score*	1.67 (2.52)	2.01 (2.61)	0.422

n = number of patients. NRS = Numerical Rating Scale for pain. * = p < 0.05. SD = standard deviation.

In the chiropractic treatment database, a strong prevalence of female patients was found for neck pain (62%) but a very slight male predominance (51%) was noted for low back pain patients. The lumbar disc herniation patients showed a very strong male prevalence of 79%.

Comparing the two genders for the imaging-guided spinal injections showed no significant differences in baseline NRS scores, 1 month NRS scores, or 1 month NRS change scores for any of the injection sites (Table 1). However, 3 of the 4 extremity injections sites (gleno-humeral (p = 0.015), subacromial (p = 0.002), knee (p = 0.023)) showed significantly higher pre-treatment (baseline) NRS scores for the female patients (Table 2). However, by 1 month there were no significant differences in the NRS scores between the genders for any of these extremity injection sites. The one month NRS change score for subacromial injections was significantly (p = 0.045) greater for the female patients.

For the patients undergoing chiropractic treatment for low back pain (n = 1065), there was a significant difference in the baseline pre-treatment NRS scores between males and females (p = 0.041) with females reporting higher scores. At 1 month the difference between the genders was higher (p = 0.001) (Table 3). Chiropractic patients with MRI confirmed lumbar disc herniations (n = 139) and patients treated for neck pain (n = 742) showed no significant differences in baseline NRS scores, 1 month NRS scores, or 1 month NRS change scores, between males and females.

**Table 3:** Gender comparisons for Pain for Chiropractic patients

**PROCEDURE**	**DATA TIME POINT**	**MALES Mean (SD)**	**FEMALES Mean (SD)**	**P-VALUE**
**Low Back Pain Chiropractic Pts (n = 1065)**	*Baseline NRS*	5.68 (2.12) n = 538	5.91 (2.27) n = 527	0.041*
	*1 Month NRS*	2.29 (2.03)	3.03 (2.33)	0.0001*
	*1 Month NRS Change score*	3.38 (2.62)	2.82 (2.83)	0.001*
**Lumbar Disc Herniation Chiropractic Pts (n = 139)**	*Baseline NRS LBP*	5.84 (2.78) n = 110	6.06 (3.27) n = 29	0.344
	*Baseline NRS Leg*	5.35 (3.18)	5.50 (3.22)	0.852
	*1 Month NRS LBP*	2.27 (1.95)	2.71 (2.23)	0.203
	*1 Month NRS Leg*	2.31 (2.26)	2.17 (2.29)	0.756
	*1 Month NRS LBP Change score*	3.39 (2.88)	3.42 (3.49)	0.728
	*1 Month NRS Leg Change score*	2.97 (2.71)	3.62 (3.08)	0.672
**Neck Pain Chiropractic Pts (n = 742)**	*Baseline NRS*	5.56 (2.10) n = 279	5.87 (2.36) n = 463	0.073
	*1 Month NRS*	2.54 (2.15)	2.83 (2.28)	0.303
	*1 Month NRS Change score*	2.98 (2.52)	2.99 (3.01)	0.666

n = number of patients. NRS = Numerical Rating Scale for pain. LBP = Low back pain. Pts = Patients. * = p<0.05. SD = standard deviation.

Assessing for significant age differences between the genders for each of the 11 musculoskeletal sites found that females were significantly older for 3 of the imaging-guided injection sites and males were significantly older for chiropractic patients treated for low back pain (Table 4). None of the other sites had significant age differences between the genders. There were no significant correlations between patient age and baseline NRS scores for any of the cohorts however.

**Table 4:** Treatment sites with statistically significant age differences between the genders

**Treatment Site**	**Males Mean age in years (SD)**	**Females Mean age in years (SD)**	**P-value**
**Epidural Injections**	55.79 (16.94)	61.44 (17.61)	0.04
**Lumbar Facet Injections**	61.07 (13.75)	64.44 (12.54)	0.009
**Knee Injections**	54.64 (12.23)	58.38 (13.88)	0.014
**Chiropractic treatment for LBP**	44.89 (14.58)	41.76 (14.36)	0.0001

SD = standard deviation. LBP = low back pain.

## Discussion

To our knowledge, this is the first study to specifically compare reported pain levels between males and females at specific anatomical sites before and after various musculoskeletal therapies using a large electronic database. Of the 11 musculoskeletal sites evaluated, females reported significantly higher baseline pain levels prior to gleno-humeral, subacromial and knee injections as well as LBP patients undergoing chiropractic treatment. However at 1 month after treatment there was no significant difference in NRS scores between the genders for the 3 extremity injection sites, indicating a better response for women. Only the LBP patients receiving chiropractic treatment continued to show the same gender difference in pain levels at one month. Although this may appear to support previous studies claiming that women report higher pain intensity compared to men [2,4,5], it also shows that only evaluating pain levels at one point in time may not be sufficient to obtain the true clinical picture, at least for some anatomical sites, and that the change in pain over time may be more important to the patient. Indeed, the female patients having the gleno-humeral, subacromial and knee injections had larger 1 month NRS change scores, which was statistically significant for the subacromial injection patients. These findings also do not support the suggestion that because females have higher baseline pain scores that they should be treated differently from males [1,3,6] nor does it agree with the findings from a recent review article stating that females tend to report higher levels of acute post-procedural pain [6], at least not for these imaging-guided injection procedures at this hospital. There were no areas in this study where male patients reported significantly higher baseline pain levels. The mechanism behind the fact that although females present with higher baseline pain levels for 4 of the 11 treatment sites, after treatment there is no gender difference in pain levels for the extremity injection patients is currently a mystery. If males have more efficient diffuse noxious inhibitory controls compared to females as reported in the literature [7,8], it would be expected that this gender difference in pain levels would persist.

Although there were statistically significant differences in baseline pain levels between the genders for 3 extremity injection sites and for chiropractic LBP patients, an important question to ask is whether or not these numerical differences are actually clinically relevant? Most previous studies reporting on gender differences in pain levels have not addressed this issue [1-6]. Indeed studies evaluating clinically meaningful pain differences have focused on the quantity of pain *reduction* levels with treatment rather than meaningful differences in pain levels between subgroups at the same time point [10-12]. It has been suggested that using the 11 point NRS, as was done in this study, a reduction of at least 1.3 points (or 20%) for patients experiencing moderate pain corresponds to ‘minimal’ improvement [11]. However, it is currently unknown if this same value can be considered the minimal meaningful difference between the genders at the same time point. In this current study the actual numerical difference between males and females for the baseline NRS scores that were statistically significantly different ranged from a low of .23 points for the chiropractic LBP patients to a maximum of .86 points for the subacromial injection patients. At least for the LBP patients it can be strongly suggested that this is not a clinically relevant pain difference between the genders. The large sex differences pain study published recently by Ruau et al. [2] reported gender differences in pain levels at one point in time for many anatomical sites and diagnoses and stated that research should look at the mechanisms behind these differences and clinicians should be paying more attention to these differences. However, a careful analysis of their data also shows small actual numerical differences between the genders for all of the areas that they reported ranging from .23 points to 1.14 points on the NRS scale. That study had a very large sample size and therefore small differences can become statistically significant but perhaps not clinically relevant.

Of the 7 different spinal sites evaluated before and after treatment, the only significant difference in outcome was for chiropractic patients treated for low back pain. Unlike previous European studies which reported that females have a higher prevalence of low back pain [4,13-17], in this current study there were slightly more males (51%) seeking chiropractic treatment for low back pain, and the male patients reported significantly less pain and more clinically relevant improvement than females at 1 month. However, no attempt was made to stratify these 1,065 low back pain patients by specific diagnosis in this study. For the low back pain patients referred for the three different imaging-guided spinal injections, it is likely that there was more homogeneity in their diagnoses, particularly for the epidural and lumbar nerve root injection patients. MRI and clinical evidence of spinal stenosis in the patients receiving epidural injections or nerve root compression in the patients undergoing nerve root blocks was typical. Interestingly, there was a higher prevalence of female patients (61%) having epidural spinal injections, and they were significantly older than their male counterparts. However, their reported pain and functional levels at baseline and 1 month after injection were no different from those reported by the male patients.

It is well established that males are more likely to have lumbar disc herniations [18,19]. However, the extremely high prevalence of 79% in the homogenous group of chiropractic patients with MRI confirmed lumbar disc herniations is most likely due to the fact that all data collected for this part of the database came from only one practice which includes three different male chiropractors. This particular practice is quite well known for treating these types of patients and the chiropractors themselves commented that their practice does attract a disproportionate number of male patients compared to other local practices [9].

The majority of neck pain patients in the chiropractic database were female (62%) as expected based on previous research [1], but for this area of the spine their baseline pain scores and outcomes were not significantly different from the male patients. As with the low back pain patients undergoing chiropractic treatment, this study also did not attempt to categorize the over 700 neck pain patients by specific diagnosis. Future studies should evaluate outcomes by gender as well as specific diagnosis. The only neck pain patients undergoing imaging-guided injections assessed in this study were those having indirect nerve root blocks. These were done if the pain was thought to arise from compression of a cervical nerve root from disc herniation, degenerative intervertebral foraminal stenosis, or a combination of the two, as determined clinically and often assisted with MRI scans. This is therefore a rather homogenous group of patients and unlike the chiropractic neck pain patients, showed a higher proportion of males (55%), consistent with the gender prevalence of cervical disc herniations reported in the literature [20]. However, there were no gender differences for pain at baseline or any of the outcomes measured at 1 month after injection.

### Limitations to the study

The main limitation to this study is the fact that only patients presenting for specific treatments involving the musculoskeletal system were included due to the availability of information from large databases. Extrapolation of these results to other anatomical sites or treatments cannot be done without additional research. Furthermore, for most of the 11 sites assessed, specific diagnoses were not easily available or confirmed. Further research into differences in reported pain levels based on gender for patients with confirmed diagnoses or specific abnormalities observed on diagnostic imaging would be valuable.

Additional demographic variables should also be considered when comparing the genders for pain differences. These could include factors such as duration of pain, ethnicity, work status, body mass index, level of physical activity and medication usage. This information was not available for most of these current patients and represents another limitation. Comparison of pain differences by age categories was also not done in this study and could potentially be relevant as increasing age has previously been shown to increase pain sensitivity [21]. However, of the 4 sites where a significant age difference between the genders was found (Table 4), only the knee injection site had a gender difference in baseline pain level. Additionally, there was no significant correlation between patient age and baseline pain scores for the 11 cohorts in this study. However, although there was no linear relationship between patient age and baseline pain levels, this does not rule out a non-linear relationship which could be a confounder for the results.

Only those anatomical treatment sites that had at least 100 patients in the database were assessed for this study. This occurred for two reasons. The first was to make sure that the sample sizes were large enough to detect any gender differences. The second reason was pragmatic as there are currently 67 different MSK injection sites with data in the radiology database, some with only a few patients. It was felt that including most of these anatomical sites in this study at this time would not add meaningful information.

Additionally, as these were all prospective outcomes studies rather than randomized clinical trials, the improvement in pain levels and overall outcomes cannot definitively be attributed to any of these treatments. Another limitation is that only change in pain at 1 month after treatment was measured rather than longer-term outcomes. However, chiropractic studies at least, have shown that if patients are likely to improve, they do so quite quickly after the start of treatment [22-24].

## Conclusions

Although 4 of the 11 musculoskeletal areas evaluated found that women had significantly higher baseline pain scores, three of which were extremity injection sites, at 1 month post-injection there were no gender differences in pain levels for these three sites. Only LBP patients receiving chiropractic treatment demonstrated statistically significant gender differences in baseline and 1 month pain levels. The actual numerical differences in the pain scores that did show statistically significant differences were not large, particularly for the chiropractic LBP cohort. It is therefore questioned whether or not these baseline gender differences in pain levels are actually clinically relevant. This shows that it may be important to measure pain levels over time, at least for certain anatomical sites, to obtain an accurate clinical picture. Although chiropractic patients suffering from neck pain were predominately women, there were no differences in baseline pain scores or any outcome measure at 1 month compared to men. It cannot be ruled out however, that the gender differences observed were due to differences in other covariates that were not measured due to their lack of availability. Future studies comparing pain levels between the genders should explore differences in adjusted models.

## Competing interests

The authors declare that they have no competing interests.

## Authors’ contributions

C K. P: Conception and design of the study; analysis and interpretation of data; drafting the manuscript. B. K H: Conception and design of the study; revising manuscript critically for important intellectual content; final approval of the version to be published. J H: Performance of imaging-guided injection procedures; revising manuscript critically for important intellectual content; final approval of the version to be published. C W. A. P: Performance of imaging-guided injection procedures; revising manuscript critically for important intellectual content; final approval of the version to be published. All authors read and approved the final manuscript.

## Pre-publication history

The pre-publication history for this paper can be accessed here:

http://www.biomedcentral.com/1471-2474/13/241/prepub
